# Diseases of the Abdomen

**Published:** 1902-11-08

**Authors:** 


### Diseases of the Abdomen.

(*Continued from page* 83.)

Dr. Ransom,[5] of Nottingham, after detailing how milk can be contaminated between the cow and the consumer, discusses the question of the relation between infantile scurvy and boiled milk. He cannot find that boiling milk produces this disease, but urges that the cause is to be looked for in milk whose consistence has been altered, or which has remained so long after sterilisation that spores have developed into bacilli; that it is a form of ptomaine poisoning, like scurvy on shipboard among adults, as pointed out by Professor Torup and others. Repeated heatings of milk to 100° C. are necessary before milk can be made spore free. He holds that cooking milk in no way interferes with its nutritive value. There is no great difference in the time of digestion of boiled or unboiled milk. Milk should be boiled, raised to the boiling point of water for 10 to 15 minutes, or Pasteurised, and if consumed before 12 hours have passed there is very slight chance of any spores having developed into bacteria. Any epidemic of summer diarrhœa should lead to an exaggeration of these precautions.

In discussing the boron compounds as preservatives, Porter[6] states that the proportion usually found in foods would exert no prejudicial effect upon the digestive ferments except perhaps rennin and amylopsin in the case of borax or boracic acid. Meat is as easily digested by gastric juice in their presence as without. A large proportion of the population show a marked idiosyncrasy with regard to boric acid manifesting deficient excretory symptoms of vomiting, diarrhœa, and skin eruptions of some severity upon the administration of medicinal doses. He considers that there is considerable unnecessary risk of obtaining a relatively toxic effect from foods preserved by such preservatives as the boron compounds, salicylic acid, formic aldehyde, sulphites, and similar antiseptics.

Duckworth and Garrod[7] relate one of those rare cases of intestinal sand. The patient was a married lady of 33 years of age. The father was gouty, and his brother and sister also. Bowel disorders were prevalent in the family. The symptoms were intractible diarrhœa, distressing extreme flatulence, and the presence of sand in the motions. No ordinary treatment availed to prevent diarrhœa and the formation of sand. Vegetable diet caused immediate distension of the bowel, and so was withheld. The patient was treated at Plombières by Dr. Bottentuit, who gave her rectal douches and the waters in small quantities, and ʒj doses of bicarbonate of sodium. Improvement followed, but there was still diarrhœa or constipation alternating; pallid and languid; no thickening of large bowel as before; and later the sand was not found. The urine showed lithates in quantity and deposit of uric acid. This case was unusual in that there was little pain, there being usually paroxysms of pain lasting several hours, like biliary or renal colic, and much flatulent distension and vomiting.

Dieulafoy regards the malady as one of the irregular expressions of the gouty state. In many cases there is coincident mucous colitis. It is more prevalent in women ($\frac{2}{3}$) than in men—the average age being 35—though it has been observed in children as young as under four years old.

The composition of the sand in this case was:—

| | |
|---|---|
| Water ... ... ... | 12·40 |
| Organic material ... ... | 26·29 |
| Inorganic material ... ... | 61·31 |
| | 100 00 |

Of this the inorganic material after combustion gave the following composition:—

| | |
|---|---|
| Calcium oxide ... ... | 54·98 |
| Phosphorus pentoxide ... | 42·35 |
| $CO_2$ ... ... ... ... | 2·20 |
| Residue containing traces of magnesium and iron ... | ·47 |
| | 100·00 |

The chief constituent would therefore seem to be calcium phosphate. The sand was of a warm brown colour with a few colourless particles, gritty to the touch, and presenting an irregular, non-crystalline, finely granular appearance under the microscope. The longest measurement was from ·05 to ·2 millimetre. Methylene-blue showed them to be rich in bacteria and micrococci. The pigment was obtained in solution by treating with acetic acid, filtering and shaking with ether. It showed a rich yellow colour, which shaken with distilled water gave a rich pink colour like acid hæamato-porphyrin, giving a broad absorption band near D. under the spectroscope, which, with the addition of hydrochloric acid, became a composite double band united by dark shading. This pink colour, even in the dark, is soon lost. Urobilin was present in a trace, and there was unaltered bile-pigment (Gmelin's test). Eichhorst has seen biliverdin in one case.

In the case recorded by Drs. Thompson and Ferguson the sand differed in size of granules and pigments. There are two distinct classes of intestinal sand—false and true. The former is composed of remains of vegetable matter undigested, such as the hard granules of pears or banana cells covered with inorganic crusts. True intestinal sand has no such vegetable basis, the organic base being of animal origin. Cases have been analysed and reported by Mathieu and Richaud, Thompson and Ferguson, Dieulafoy, Marquez, Biaggi, and Mongour. The origin of the sand is not biliary, as no cholesterin is present, but is with very little doubt formed in the intestine itself.

[5] Brit. Med. Jour., Feb. 22. [6] Brit. Med. Jour., May 10
[7] Lancet, March 8.

*(To be continued.)*

